# Nutritional and Age-Related Challenges in Older Adults from Sub-Saharan Africa and Potential Strategies to Promote Healthy Aging Amongst Them: A Systematic Review

**DOI:** 10.3390/nu18091346

**Published:** 2026-04-24

**Authors:** Vanessa Adu Sarpong, Isaac Amoah, Mauro Lombardo, Phyllis Tawiah, Wenze Wu, Kate Ampomah Addo, Deborah Solomon

**Affiliations:** 1 Department of Biochemistry and Biotechnology, Kwame Nkrumah University of Science and Technology, Kumasi 00233, Ghana; vadusarpong@st.knust.edu.gh (V.A.S.); kaaddo8@st.knust.edu.gh (K.A.A.); dsolomon@st.knust.edu.gh (D.S.); 2 Department for the Promotion of Human Science and Quality of Life, San Raffaele Open University, Via di Val Cannuta, 247, 00166 Rome, Italy; mauro.lombardo@uniroma5.it; 3 Department of Medicine, School of Medicine and Dentistry, College of Health Sciences, Kwame Nkrumah University of Science and Technology, Kumasi 00233, Ghana; phytawiah@knust.edu.gh; 4 Inner Mongolia Key Laboratory of Life Health and Bioinformatics, School of Life Science and Technology, Inner Mongolia University of Science and Technology, Baotou 014010, China; wuwenze@alu.sxu.edu.cn; 5 College of Life and Health Sciences, Northeastern University, Shenyang 110819, China

**Keywords:** dysphagia, frailty, geriatrics, malnutrition, sarcopenia

## Abstract

**Background/Objectives**: Aging is associated with physiological, biochemical, and psychosocial changes that can significantly affect nutritional status and overall health. In Sub-Saharan Africa (SSA), older adults face unique age-related challenges that may compromise healthy aging, yet evidence remains fragmented. This systematic review synthesized the existing literature on the nutritional status, age-related challenges, and strategies to promote healthy aging of older adults in SSA. **Methods**: A systematic literature search was conducted on PubMed, Scopus, ScienceDirect, and Cochrane Library to identify relevant studies published up to 10 December 2025. **Results**: Fifty-five studies met the inclusion criteria, with most of the studies coming from South Africa, Ghana, and Nigeria. Amongst community-dwelling populations, approximately 30–65% of the older adults were either malnourished or at risk of malnutrition, while hospital-based studies reported markedly higher burdens, with malnutrition prevalence exceeding 70% in some settings. Undernutrition, micronutrient deficiencies, and the coexistence of overweight and obesity were frequently observed, reflecting the region’s ongoing nutrition transition. Frailty emerged as the predominant age-related challenge, with prevalence ranging around 10–60%. Other common challenges included sarcopenia, reduced muscle strength, functional disability, cognitive impairment, and dysphagia, all of which were closely related to poor nutritional status, food insecurity, multimorbidity, and reduced quality of life. Few studies reported on healthy aging strategies, with the limited evidence suggesting that nutrition education, physical activity, and psychosocial interventions may enhance nutritional and functional outcomes. **Conclusions**: The need for context-specific, nutrition-sensitive interventions, and stronger health and social support systems is warranted to promote healthy aging in SSA older adults.

## 1. Introduction

In several developed countries of the world, a demographic shift characterized by a surge in the proportion of older adults and a decline in population growth due to lower birth rate is currently taking place [1,2]. Africa, however, presents a different scenario in its population demographics. Despite the continent being regarded as one of the youngest in the world, Africa has recorded an increased growth in the proportion of older adults in recent times [3,4,5]. For example, between 1960 and 2005, the life expectancy of Africans in the Sub-Saharan region was 54 years. This however increased to 59 and 63 years, respectively, between 1960 and 2015 and between 1960 and 2023, showing a steady growth in the number of older adults in the region [6]. Improvements in public health services, ease of access to healthcare facilities, availability of vaccines, adherence to regular medical check-ups, and gradual epidemiological transition have contributed to the increased life expectancy observed in Sub-Sahara Africa (SSA) [7]. The increased proportion of older adults presents a significant burden on health systems in SSA, where historically medical care had focused on the management of infectious diseases [8,9].

Aging presents several nutrition-related challenges, including dysphagia, sarcopenia, protein malnutrition, and frailty, which occur as a result of the biochemical and physiological changes that takes place in the body [10,11,12,13]. Nutrition plays a fundamental role in determining the health, functional capacity, and quality of life of older adults [14]. According to Wickramasinghe et al. [15], healthy aging is closely linked to adequate nutritional status, which supports physiological resilience, immune competence, cognitive function, and independence. Conversely, malnutrition, encompassing undernutrition, micronutrient deficiencies, and the emerging burden of overweight and obesity [15], remains a critical yet under-recognized challenge among older Africans.

Evidence from SSA suggests that the magnitude of malnutrition among older adults varies widely across settings, reflecting diverse socioeconomic and cultural contexts [16]. Several studies have reported substantial prevalence of undernutrition in rural and resource-poor communities, while overweight and obesity are increasingly observed in urban communities undergoing nutrition transition [17,18,19,20]. Micronutrient deficiencies, particularly calcium, vitamin D, and iron deficiencies, and inadequate protein are also common nutrient deficiencies in older adults and are often linked to frailty, impaired mobility, and increased morbidity [21]. Importantly, age-related conditions such as sarcopenia and functional limitations are frequently reported to be associated with poor diet quality and inadequate protein intake, further exacerbating vulnerability in older populations [22]. Yet, despite these patterns, research on the nutritional status of older adults in SSA remains fragmented and geographically uneven [16,23].

Strategies to promote healthy aging through improved nutrition have gained increasing global attention; however, evidence from SSA remains limited and less systematically developed compared with high-income regions [8,24]. Existing interventions in SSA include community-based nutrition education, food supplementation programs, social protection initiatives such as cash transfers and pensions, and integrated primary healthcare models targeting older adults [25,26,27]. Nevertheless, many of these interventions have not been rigorously or systematically evaluated, and context-specific evidence on their effectiveness, sustainability, and acceptability among older populations remains scarce [23,28]. Furthermore, the limited availability of geriatric nutrition guidelines and the absence of standardized nutrition screening and assessment tools within routine clinical practice continue to constrain the delivery of effective and age-appropriate nutrition care for older adults in the region [29,30].

Given the rising population of older adults and the complex interplay between aging, nutrition, and health outcomes, there is a clear need for a comprehensive synthesis of the available evidence [31]. This systematic review aims to consolidate current knowledge on the nutritional status of older adults in SSA, the age-related challenges influencing their nutritional well-being, and potential strategies implemented to support healthy aging in this demographic. By examining prevalence patterns, determinants, associated health consequences, and intervention approaches, this review seeks to highlight critical gaps in the literature and guide future research, policy development, and program implementation. This review synthesizes the existing literature on the nutritional status of older adults in SSA, age-related challenges, and potential strategies to promote healthy aging.

## 2. Materials and Methods

**Figure 1:**
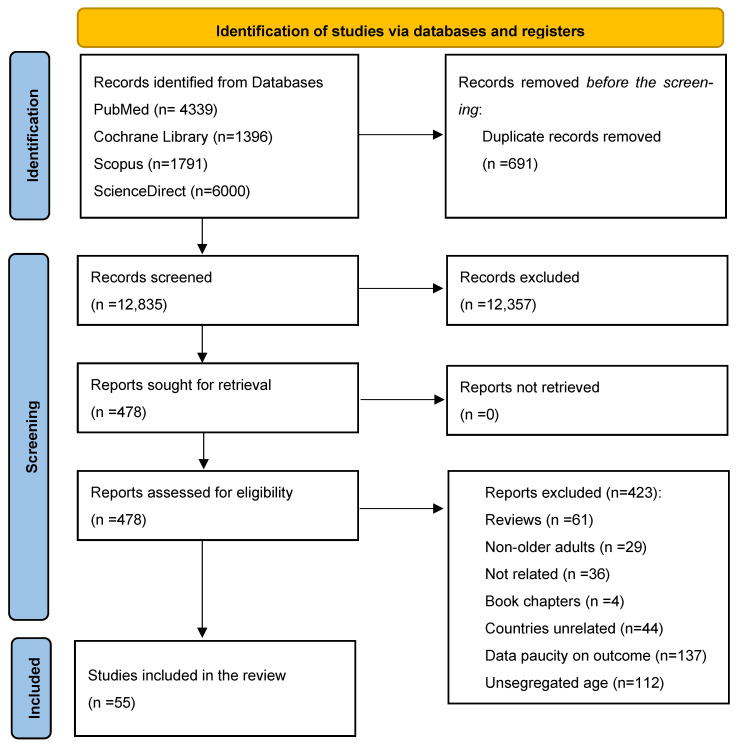
Flowchart summarizing studies evaluated and selected for our systematic review.

This systematic review was conducted and reported in accordance with the guidelines of the recently updated Preferred Reporting Items for Systematic Reviews and Meta-Analysis (PRISMA) statement [32] (Figure 1). A systematic search was conducted to identify studies on nutritional status, age-related challenges, and strategies to promote healthy aging among older adults in Sub-Saharan Africa using the databases PubMed, Cochrane Library, ScienceDirect, and Scopus on 10 December 2025.

The search terms employed on PubMed and Cochrane Library included the following: (nutritional challenge* OR nutritional status OR nutrition OR nutrition problem OR age-related challenge* OR dysphagia OR malnutrition OR sarcopenia OR frailty) AND (older adult* OR elderly OR older person* OR aged) AND (Sub-Saharan Africa* OR Sub Saharan Africa* OR Ghana OR Nigeria OR Zambia OR Ethiopia OR Angola OR Benin OR Botswana OR Burkina Faso OR Burundi OR Cameroon OR Cape Verde OR Central African Republic OR Chad OR Comoros OR Congo OR Congo (Democratic Republic) OR Côte d Ivoire OR Djibouti OR Equatorial Guinea OR Eritrea OR Gabon OR The Gambia OR Guinea OR Guinea-Bissau OR Kenya OR Lesotho OR Liberia OR Madagascar OR Malawi OR Mali OR Mauritania OR Mauritius OR Mozambique OR Namibia OR Niger OR Réunion OR Rwanda OR Sao Tome and Principe OR Senegal OR Seychelles OR Sierra Leone OR Somalia OR South Africa OR Sudan OR Swaziland OR Tanzania OR Togo OR Uganda OR Western Sahara OR Zimbabwe). Search terms for ScienceDirect and Scopus included: (nutritional challenge OR nutritional status OR age-related challenge OR dysphagia OR malnutrition OR sarcopenia) AND (older adult OR elderly) AND (Sub-Saharan Africa).

The eligibility criteria for study selection were defined a priori to ensure methodological rigor and relevance to the review objectives. Studies were included if they were (i) original research articles; (ii) conducted among older adults, typically defined as individuals aged 50 years and above (this definition is consistent with the World Health Organization’s (WHO) recommendations for low- and middle-income countries, where life expectancy is generally lower than in high-income settings and also corresponds with the criteria used in the WHO Study on global AGEing and adult health (SAGE), a longitudinal study conducted in Ghana, South Africa, Mexico, India, China, and Russia [33]); (iii) written in English, with its full text readily accessible; (iv) conducted within Sub-Saharan Africa; and (v) addressing at least one of the following domains in older adults—nutritional status, age-related health challenges (including frailty, sarcopenia, and dysphagia), or strategies aimed at promoting healthy aging.

Studies were excluded if they were (i) review articles, conference proceedings, letters to editors, commentaries, or book chapters; (ii) studies that did not involve older adults; (iii) those in which age-specific data could not be isolated; (iv) studies conducted outside the Sub-Saharan African region; and (v) studies that were not directly related to the thematic focus of nutritional status and healthy aging, or those with insufficient data on key outcomes of interest.

Study characteristics extracted were the authors’ names and year of publication, country of study, study design employed, sample size, characteristics of the participants, objective of study, outcomes of assessment, and findings of the study.

Three independent authors, V.A.S., K.A.A., and I.A., were involved with the searching and selection of the articles. In instances where there was disagreement between the authors, a consensus was established through discussion with authors I.A. and P.T. The articles were exported to the reference manager ENDNOTE™ Library. No filters were applied to searches on PubMed and Cochrane Library, but filters were applied to ScienceDirect and Scopus to restrict to only original research articles.

A methodological quality appraisal of all included studies was conducted using design-specific Joanna Briggs Institute (JBI) Critical Appraisal Checklists. Studies were first grouped according to their respective study designs, that is, observational [34], cohort [35], quasi-experimental [36], and qualitative [37] study designs, and the appropriate JBI tool was applied to each category. Each domain in the checklist was rated as “yes”, “no”, or “unclear”, depending on whether the criterion was met, not met, or insufficiently reported.

## 3. Results

### 3.1. Study Selection

A total of 13,526 records were retrieved from the databases PubMed, Cochrane Library, ScienceDirect, and Scopus. Following the removal of 691 duplicate records, a total of 12,835 records were obtained. The 12,835 records were screened for their titles and abstracts, resulting in 478 records that were assessed for eligibility by applying the inclusion and exclusion criteria. A total of 55 study reports were identified as eligible and included in this systematic review. A detailed description of the search and study selection process is summarized in Figure 1.

### 3.2. Geographical Distribution of the Selected Studies

The distribution of studies across countries revealed a strong geographical concentration, with notable disparities both at the country and sub-regional levels. Ghana (n = 14) and South Africa (n = 12) accounted for the highest number of studies, followed by Nigeria (n = 8), Ethiopia (n = 6), and Tanzania (n = 6). Uganda contributed four studies, while Côte d’Ivoire accounted for three. Cameroon and Democratic Republic of Congo were each represented by two studies. The remaining countries, that is, Malawi, The Gambia, Kenya, Lesotho, Rwanda, and the Central African Republic, were each represented by a single study.

When disaggregated by sub-region, West Africa contributed the largest share (n = 26), largely driven by Ghana and Nigeria, followed by East Africa (n = 18), where Ethiopia and Tanzania predominated. Southern Africa accounted for 14 studies, primarily due to South Africa, whereas Central Africa was markedly underrepresented, contributing only five studies across four countries. This pattern highlights a significant concentration of evidence in few countries and sub-regions, with comparatively limited representation from much of Central Africa. Multi-country studies were disaggregated and accounted for each country represented, resulting in a total of 63 country occurrences (Table 1, Table 2, Table 3, Table 4 and Table 5).

Study setting was inconsistently reported across the included studies. Among the studies that provided information on study settings (n = 38), there was a clear predominance of participants drawn from urban and peri-urban settings. Specifically, 24 studies were conducted exclusively in urban areas, including major cities and regional capitals, while nine studies included mixed urban–rural populations. In contrast, only five studies explicitly reported on rural populations. However, several community-based studies did not clearly specify the rural–urban composition of their samples, suggesting that rural representation may be underestimated. Reporting of socioeconomic characteristics was similarly heterogeneous and often relied on proxy indicators rather than standardized measures. Approximately 21 studies described participants as belonging to low-income groups or relying on informal employment, while 15 studies reported indicators suggestive of limited access to healthcare services. Additionally, 12 studies referenced aspects of household food insecurity or low dietary diversity, particularly among older adults in economically constrained settings. However, standardized socioeconomic measures such as income quintiles, asset-based indices, or educational gradients were reported in fewer than 10 studies, thereby limiting comparability across studies and contexts.

### 3.3. Classification of Malnutrition Prevalence by Assessment Tool and Setting

Malnutrition prevalence varied substantially across studies, reflecting differences in assessment tools, study settings, and population characteristics, rather than a single underlying pattern. As presented in Table 2, studies employing different measurement approaches reported markedly different estimates, underscoring the influence of methodological variation on observed outcomes. Studies utilizing the Mini Nutritional Assessment (MNA) tool and its short form (MNA-SF) generally reported both malnutrition and “at risk” categories, providing a more nuanced classification of nutritional status. Within this group, prevalence varied widely across settings. For example, Corso et al. [40] reported that in Ghana, fewer than 10% of older adults were malnourished, while 48.9% were at risk, compared to 3.6% malnourished and 31.3% at risk in South Africa. Similarly, Naidoo et al. [50] reported 5.5% malnutrition and 43.4% at risk among community-dwelling older adults, while Isong et al. [44] observed higher vulnerability in more dependent populations, with 9.1% malnourished and 58.2% at risk. In contrast, higher estimates were reported in hospital-based populations, such as in the study by Mphwanthe et al. [49], where 40.3% of participants were malnourished and 39.7% at risk, and Tesfaye et al. [53], who reported 81.0% malnutrition among in-patients. These findings suggest that clinical populations consistently exhibit a higher burden of malnutrition when assessed using standardized tools.

Studies based on anthropometric measures (such as BMI, MUAC, and other body composition indices) also demonstrated considerable variability. Community-based studies such as those by Abdu et al. [38] and Dufe Turkson [41] reported relatively lower malnutrition prevalence (15.7% and 14.6%, respectively), although a large proportion of participants were classified as at risk (51.7% and 66.0%). Similarly, Jésus et al. [45] reported an undernutrition prevalence of 19.2% across Central African Republic and the Republic of Congo, while Mabiama et al. [47] found 19.7% undernutrition in Cameroon. In contrast, hospital-based anthropometric assessments yielded substantially higher estimates, with Okoro et al. [51] reporting 71.3% malnutrition among hospitalized older adults and Olawumi et al. [52] reporting 25.3% malnutrition alongside 56.6% at risk. These findings further highlight the influence of clinical status and care setting on nutritional outcomes.

### 3.4. Classification of Frailty Prevalence by Assessment Tool and Setting

Frailty prevalence was examined by explicitly stratifying studies according to assessment tool and study setting, given the substantial variation in frailty definitions and measurement approaches (Table 4). This stratification is critical, as prevalence estimates are not directly comparable across tools that operationalize frailty differently. Studies employing deficit accumulation approaches, such as the 30- or 40-item Frailty Index, consistently reported higher prevalence estimates, particularly in broader population or mixed settings. For example, Ambagtsheer and Moussa [56] reported a frailty prevalence of 60.0% in Côte d’Ivoire using a 30-item Frailty Index, while Dzando and Moussa [64] similarly reported 59.3% using the same approach. Biritwum et al. [60] also reported a prevalence of approximately 38% across Ghana and South Africa using a 40-item Frailty Index. These higher estimates likely reflect the cumulative nature of deficit-based tools, which capture a wide range of health deficits.

In contrast, studies using phenotype-based measures, particularly the Fried Frailty Phenotype, generally reported lower prevalence estimates, especially in community-based populations. Lewis et al. [75] reported frailty prevalence ranging from 9.3% to 11.2% among community-dwelling older adults in Tanzania, while Mbabazi et al. [79] reported prevalence of 13.5–15.1% in Ugandan outpatient populations. Similarly, Mwangala et al. [80], using the Edmonton Frail Scale, reported a prevalence of 24% among older adults living with HIV in Kenya, reflecting intermediate estimates associated with multidimensional screening tools. Stratification by study setting further highlights important differences. Lower prevalence estimates were typically observed in community and outpatient settings, whereas substantially higher estimates were reported in hospital-based populations, where participants are more likely to have acute illness and multiple comorbidities. For instance, Davidson et al. [63] reported frailty prevalence of 66.6% using the Clinical Frailty Scale and 57.0% using the Frailty Phenotype among hospitalized older adults in Tanzania.

Importantly, variability was also evident within the same population depending on the assessment tool applied, underscoring the lack of interchangeability between frailty measures. Bristow et al. [62] reported frailty prevalence ranging from 0.68% using the Clinical Frailty Scale and B-FIT tool to 2.7% using the Fried phenotype in a rural Tanzanian cohort, demonstrating the sensitivity of prevalence estimates to measurement approach. Furthermore, some tools, such as the Tilburg Frailty Indicator and locally adapted instruments like FiSSA, report continuous frailty scores rather than categorical prevalence, as seen in studies by Asiamah et al. [57] and Kasa et al. [22], further limiting comparability.

### 3.5. Quality Assessment and Scoring of Methodological Quality

The methodological quality of included studies was assessed using design-specific Joanna Briggs Institute (JBI) critical appraisal tools appropriate to each study design. Each checklist item was rated as “yes”, “no”, or “unclear”, where “unclear” indicated insufficient reporting to determine whether the criterion was met. To enhance transparency and reproducibility, an explicit scoring approach was applied to derive overall study quality. For each study, the number of “yes” responses was summed and expressed as a proportion of the total applicable items. Studies were then categorized as high quality (≥70% of items rated “yes”), moderate quality (50–69%), or low quality (<50%). “Unclear” and “no” responses were not counted as meeting the criterion. All checklist items relevant to each study design were included in the assessment, and no domains were omitted. Quality appraisal was conducted independently using these predefined criteria to ensure consistency and minimize subjectivity. The detailed results of the quality appraisal, including item-level ratings, are presented in Table 6, Table 7, Table 8 and Table 9.

## 4. Discussion

In this present systematic review, Ghana, Nigeria, Ethiopia, South Africa, and Tanzania were the countries that reported the highest number of studies. This is not surprising, as Ghana and South Africa, for example, were the only two African countries included in one of the most comprehensive longitudinal studies in older adults aged 50 years and above carried out by the World Health Organization (WHO), titled the “WHO Study on global AGEing and adult health (SAGE)”, which involved other countries such as Mexico, Russia, China, and India [33]. The SAGE study has contributed to an excellent publication output in the geriatric space with Ghanaian and South African older adults as participants [40,61,81,82,91,92,93]. Additionally, several major research centers have been established in Ghana, Nigeria, Ethiopia, South Africa, and Tanzania and have integrated aging-related research into their core priorities. These research priorities tie in with the national objectives of these countries, where, in some cases, funding grants may be provided by the central governments to strengthen research capacities in those areas [91]. The higher research outputs in the geriatrics and gerontology area evidenced from this review is a show of active research work from these centers. Examples of these research centers include the West African Centre for Cell Biology of Infectious Pathogens (Ghana) [94]; Centre for Ageing Studies (Ghana) [95]; Research Center for Ageing Cognition and Psychological Health (Nigeria) [96]; Applied Gerontology Research Group (Nigeria) [97]; Albertina and Walter Sisulu Institute of Ageing in Africa (South Africa) [98]; International Longevity Centre—South Africa (ILCSA) [99]; Ageing and Generational Dynamics in Africa (AGenDA) (South Africa) [100]; Kilimanjaro Clinical Research Institute (KCRI) (Tanzania) [101]; Ethiopian Public Health Institute (EPHI) [102]; and Aide for Ghanaian Elderly (AGE) [103].

Additionally, the increased research activities in several of the SSA countries highlights the ongoing demographic transition in the region, where improvements in healthcare and living conditions have contributed to an increasing life expectancy and a steadily expanding older adult population [104]. Although these factors may plausibly influence the observed distribution of studies in the broader literature, the present synthesis was not designed to investigate such determinants. Life expectancy in SSA remains relatively lower than that reported in developed countries. Recent estimates indicate notable improvements, with several African countries now recording life expectancies exceeding 60–65 years. This demographic shift has been accompanied by a rising burden of age-related nutrition problems, chronic diseases, functional decline, and frailty, necessitating increased research attention [105,106].

Across the included studies, participant characteristics showed consistent patterns that are critical for interpreting the observed nutritional and aging-related outcomes among older adults in Sub-Saharan Africa (SSA). Most studies focused on community-dwelling older adults aged 60 years and above, with average participant ages typically falling between 60 and 70, whereas adults aged 80 years and above were underrepresented [38,60,76]. Women constituted a larger proportion of participants across studies, reflecting longer female life expectancy, and being of female gender was frequently associated with poorer nutritional status, higher risk of developing frailty, and functional limitation [41,54,65]. Socioeconomic vulnerability was widespread, with many participants reporting low educational attainment, low income, and financial dependence, and food insecurity was common where assessed, highlighting its central role in impacting nutritional status in later life [21,39,49].

Health and functional characteristics further underscored the vulnerability of the study populations. A large proportion of participants lived with one or more chronic conditions, including hypertension, diabetes mellitus, cardiovascular disease, HIV, and chronic respiratory illnesses, with multimorbidity often associated with malnutrition, frailty, and reduced quality of life [40,46,52]. Hospital-based populations, though fewer in number, consistently exhibited more severe nutritional deficits and functional impairment than community-based samples [51,53]. Low levels of physical activity were frequently reported and were associated with poorer nutritional and functional outcomes [72,88], while sensory impairments, pain, sleep disturbances, and depressive symptoms further compounded vulnerability among older adults [59,73,78]. Collectively, these participant characteristics depict older adults in SSA as a heterogeneous yet highly disadvantaged population, underscoring the need for gender-sensitive, context-specific, and equity-oriented interventions to address malnutrition and promote healthy aging.

The findings of the review demonstrate that malnutrition and risk of malnutrition are highly prevalent among older adults in Sub-Saharan Africa (SSA), though the magnitude varies considerably by setting, population group, and assessment method (Table 1 and Table 2). This is supported by the fact that global evidence indicates that aging is associated with physiological changes that compromise dietary intake, nutrient absorption, and metabolic efficiency [107,108]. However, the magnitude of malnutrition observed in SSA appears substantially higher than that reported in high-income countries, where community-dwelling older adults are shown to typically exhibit malnutrition prevalence rates below 30% [109]. Across community-based studies, between one-third and two-thirds of older adults were either malnourished or at risk of malnutrition, underscoring the scale of the problem in this population [38,41,50]. This wide variability reflects heterogeneity in socioeconomic conditions, food systems, health status, and access to care across SSA, as well as differences in nutritional assessment tools used across studies.

Hospital-based studies consistently reported more severe forms of malnutrition compared with community-dwelling populations. For example, extremely high prevalence rates of malnutrition were observed among hospitalized older adults in Ethiopia and Nigeria, with over 70% classified as malnourished in some settings [51,53]. This pattern aligns with existing evidence that hospitalization in older age is often associated with worse nutritional status, likely reflecting the cumulative effects of acute illness, reduced appetite, functional impairment, and polypharmacy, which further exacerbate pre-existing nutritional deficits [39,49]. The observed heterogeneity across studies reflects differences in socioeconomic context, disease burden, dietary patterns, and nutritional assessment tools, underscoring the structural vulnerability of older adults in SSA [110,111]. Consequently, older adults in SSA may enter old age after a lifetime of exposure to food insecurity, infectious diseases, and limited healthcare access, which can compromise their nutritional reserves, rendering them particularly vulnerable to malnutrition when confronted with illness or socioeconomic stressors [112].

According to Table 2, differences in study setting were a major driver of variation in malnutrition across all assessment methods. Lower prevalence estimates in malnutrition were generally observed in community-dwelling populations, while significantly higher prevalence was consistently reported in hospital or clinical settings, where participants are more likely to be presented with comorbidities, functional impairment, and other illness. Additionally, population-specific factors, such as chronic conditions (e.g., hypertension and diabetes), dependency status, and socioeconomic constraints, were frequently identified as contributors to increased malnutrition risk. Importantly, substantial variation was also observed within similar populations depending on the assessment tool used, indicating that measurement approach plays a critical role in determining prevalence estimates. Furthermore, some studies reported only malnutrition or risk categories without full classification, while others used different thresholds or indicators, limiting direct comparability. Taken together, these findings demonstrate that the observed variability in malnutrition prevalence is primarily driven by methodological and contextual differences, including assessment tools, study settings, and population characteristics. Therefore, these estimates should not be interpreted as directly comparable or combined into a single summary range. Instead, they should be understood as context-specific indicators of nutritional status within distinct study conditions, highlighting the need for standardized assessment approaches in future research.

The coexistence of undernutrition, overweight, and obesity among older adults observed in this review reflects the broader nutrition transition occurring across low- and middle-income countries [113,114]. Similar patterns have been reported globally, where shifts toward energy-dense but nutrient-poor diets contribute to the paradoxical presence of obesity alongside micronutrient deficiencies, particularly among socioeconomically disadvantaged populations. In SSA, this transition is often characterized by reliance on inexpensive refined staples and limited intake of high-quality protein, fruits, and vegetables, which may explain the persistence of malnutrition even among individuals with excess body weight [114]. Similar trends have been reported in Latin America and parts of Asia, reinforcing that excess body weight does not preclude nutritional risk, particularly in older populations [19].

Chronic diseases emerged as a critical determinant of nutritional status. Studies involving older adults with heart failure, diabetes mellitus, or multimorbidity consistently reported higher levels of undernutrition, underweight, and nutritional risk compared with healthier counterparts [39,46,52]. The coexistence of overweight, obesity, and undernutrition, particularly among individuals with diabetes, highlights the double burden of malnutrition affecting older adults in SSA [46,47]. This phenomenon reflects ongoing nutrition transition, especially in urban and peri-urban areas, where energy-dense but nutrient-poor diets coexist with persistent food insecurity.

Food insecurity was also strongly linked to poor nutritional and functional decline among older adults in SSA. This finding is consistent with studies from both high- and low-income settings, demonstrating that food insecurity can be associated with malnutrition, disability, and poorer quality of life in later life [115]. Evidence from Ghana demonstrated that older adults reporting insufficient food intake or hunger were significantly more likely to experience functional decline [21]. This finding emphasizes that malnutrition among older adults in SSA is not solely a biomedical issue but is deeply embedded in broader socioeconomic constraints, including poverty, limited income security, and weak social protection systems.

Also, studies assessing diet quality and protein intake highlighted important protective factors. In Rwanda, consumption of animal-sourced foods was associated with higher protein intake and greater muscle mass, reinforcing the importance of dietary diversity in maintaining functional health in later life [43]. Similarly, longitudinal evidence from Ghana and South Africa showed that better nutritional status was associated with improved cognition and grip strength over time, confirming the long-term functional implications of adequate nutrition in older age [40].

This review demonstrates that aging-related challenges among older adults in SSA are multidimensional and influenced by one’s nutritional status (Table 3). Frailty emerged as the most frequently reported condition, with prevalence estimates ranging from about 10% in some community-dwelling older adults to over 60% in hospital-based and low-income populations [56,63]. The reported prevalence of frailty varied substantially across studies, reflecting differences in assessment tools, study settings, and population characteristics. These estimates are comparable to, and in some cases higher than those, reported in other low-resource settings globally, but they exceed the prevalence rates observed in high-income countries [116]. The findings in Table 4 further indicate that differences in frailty definitions, assessment tools, and study settings are the primary drivers of variability in reported prevalence. As such, presenting frailty as a single range across studies would be misleading. Instead, prevalence estimates should be interpreted within the context of specific measurement approaches and population settings, highlighting the need for greater standardization in frailty assessment in Sub-Saharan Africa.

Frailty was consistently higher among women and increased markedly with age, reflecting a biological and lifelong socioeconomic disadvantage [60,64]. The association between frailty and poor nutritional status observed across multiple studies aligns with existing conceptual models that position malnutrition as both a driver and outcome of frailty [22,117]. Similar bidirectional relationships have been documented in European and Asian cohorts, where inadequate protein and energy intake contribute to muscle loss, reduced strength, and functional decline, which in turn impair food intake and dietary adequacy [118,119].

Sarcopenia and reduced muscle strength were also prominent challenges, with prevalence estimates ranging from approximately 12% to 27% depending on the population and diagnostic criteria. Low grip strength and slow gait speed were repeatedly associated with functional disability, poorer self-rated health, and cognitive decline [54,61,74]. These findings reinforce the critical role of nutrition, particularly adequate protein and energy intake, in preserving muscle mass and physical function in older adults. The observed associations between poor nutritional status, frailty, and sarcopenia further highlight the bidirectional relationship between nutrition and aging-related decline [22].

Mental health and cognitive challenges further compounded nutritional vulnerability. Depression, emotional pain, sleep disturbances, and cognitive impairment were frequently reported and often coexisted with physical frailty and chronic disease [55,71,78]. Similar associations have been widely reported in the global aging literature, where depression and cognitive impairment were known to reduce appetite, disrupt meal preparation, and impair dietary adherence [120,121]. Longitudinal evidence from Nigeria and Tanzania showed substantial prevalence of dementia and mild cognitive impairment among the older populations, conditions that can further impair food intake, dietary adherence, and self-care capacity [58,74].

Sensory and functional impairments such as dysphagia, hearing loss, pain, and mobility limitations further exacerbate the nutritional risk in this population. Swallowing difficulties were common among institutionalized older adults and were associated with increased effort during eating and medication intake, potentially leading to reduced food consumption and weight loss [59]. Evidence from South Africa indicated that nearly half of institutionalized older adults reported swallowing difficulties, which may substantially reduce food intake and increase the risk of malnutrition [59]. Comparable studies from Europe and Asia have similarly identified dysphagia as a major determinant of undernutrition in older populations, particularly in long-term-care settings [122,123]. Similarly, bodily pain and sleep problems were independently associated with poorer self-rated health and emotional distress, factors that may indirectly worsen appetite and dietary quality [71].

Across the included studies, malnutrition, frailty, and sarcopenia were frequently reported to be associated; however, these relationships are complex and potentially bidirectional, and so they should be interpreted with caution. Poor nutritional status may contribute to the development and progression of frailty and sarcopenia through mechanisms such as reduced protein intake and muscle mass loss; meanwhile, these conditions may in turn impair dietary intake, appetite, and functional ability, thereby exacerbating malnutrition [124,125,126]. Importantly, the majority of studies included in this review were observational, predominantly cross-sectional, and therefore limited in their ability to establish causal pathways. Furthermore, although consistent patterns of association were observed, these findings should not be interpreted as evidence of causality, as more robust longitudinal and interventional studies are needed to clarify these complex relationships.

Reported strategies and associated factors to promote healthy aging in SSA remain limited, fragmented, and largely observational, with few rigorously evaluated interventions (Table 5). In this review, “healthy aging strategies” were defined as interventions or modifiable factors that influence nutritional status, functional capacity, or quality of life in older adults [127]. Nutrition-specific interventions were notably scarce in Table 5. Most studies did not evaluate formal interventions but rather examined associations between lifestyle or socioeconomic factors, such as physical activity, income status and living conditions, and quality of life or health outcomes. Only two (n = 2/55) studies employed an interventional design. Only one pre–post-study evaluated the effect of nutrition counselling and reporting significant improvements in nutritional knowledge without corresponding changes in anthropometric outcomes [90]. This finding suggests that knowledge alone may be insufficient to improve nutritional status in settings where food access, affordability, and health constraints limit dietary change [86]. Frailty consistently emerged as a key determinant of poorer QoL, reinforcing the need for early identification and management of nutritional and functional decline to support healthy aging trajectories.

Quality of life (QoL) was the most commonly assessed outcome, with multiple studies demonstrating strong associations between physical health, income security, functional ability, and perceived well-being [66,85]. Among older adults living with HIV, improvements in perceived QoL following initiation of antiretroviral therapy further underscore the role of integrated health services in supporting healthy aging [84]. These findings align with evidence from high-income and middle-income countries, demonstrating that active aging and social engagement are critical determinants of healthy aging trajectories [128].

Age-related physiological changes, oral health problems, neurological conditions, and frailty frequently impair mastication and swallowing, leading to reduced food intake; dietary monotony; and avoidance of nutrient-dense foods such as meats, fruits, and vegetables [129]. Nutritional approaches are often tailored to develop texture-modified, high-quality, nutrient-dense foods to improve dietary intake, nutritional adequacy, and functional health outcomes among older adults, particularly those experiencing chewing and swallowing difficulties [16,130]. Texture modification of the food matrix aims to improve the safety and ease of swallowing, thereby supporting adequate oral intake and reducing the risk of aspiration, choking, and mealtime distress [130,131]. The International Dysphagia Diet Standardisation Initiative (IDDSI) provides a globally harmonized framework for classifying foods and fluids according to texture and thickness, enabling consistent prescription and implementation of texture-modified diets across clinical and community settings [131]. By aligning food textures with an individual’s functional swallowing capacity, IDDSI-guided approaches help ensure that meals remain both safe and acceptable for older adults with dysphagia [130].

Reformulation strategies such as incorporating legume flours, dairy or dairy alternatives, fish powders, eggs, and plant-based protein isolates into familiar staples (e.g., porridges, breads, dumplings, and composite flours) could substantially improve protein density without increasing meal volume [132,133,134,135]. These strategies aim to enhance protein and micronutrient intake, support the prevention of sarcopenia, and reduce the risk of malnutrition while maintaining food acceptability and safety [136,137,138]. They also aim to optimize nutrient absorption and improve overall nutritional status, thus reducing morbidity and mortality risks in older adults across Sub-Saharan Africa [16].

Lifestyle-related factors, including physical activity and autonomy over household resources, were consistently linked to better health outcomes. Older adults who controlled household assets or engaged in regular physical activity were significantly more likely to report good physical health, highlighting the importance of the economic empowerment and active aging frameworks in SSA contexts [88]. The World Health Organization recommends that older adults engage in 150 to 300 min of moderate-intensity aerobic activity, including brisk walking, dancing, and gardening, per week, alongside muscle-strengthening exercises at least twice weekly and balance-enhancing activities for those with mobility concerns [139].

Education on the benefits of exercise, integration into routine healthcare, and monitoring improvements with simple functional assessments could further enhance participation [140]. Evidence from some SSA studies indicate that physical-activity participation tends to be lower with increasing urbanization and sedentary lifestyles, yet walking, dancing, and group-based activities are commonly preferred and culturally acceptable forms of exercise among older populations [141,142,143]. In addition to physical benefits, engagement in even light physical activity is often associated with reduced pain and improved musculoskeletal outcomes, further illustrating the multidimensional relevance of activity promotion in aging contexts [144,145,146]. Physical activity, therefore, represents a low-cost, high-impact approach that can prevent frailty, chronic diseases, and functional decline while promoting overall health and well-being among older adults in the region.

According to Liu and Wang [147], social isolation, often exacerbated by shifts in post-retirement lifestyles and diminishing social circles, can profoundly impact an individual’s social support network, leading to mental health challenges and diminished quality of life [148]. Furthermore, interventions focusing on improving self-efficacy, morale, and quality-of-life perceptions through structured programs have demonstrated beneficial effects on psychosocial well-being [149]. Engagement in long-distance travel has been reported to improve cognitive functioning, alongside lowering the prevalence of depressive symptoms and diminishing experiences of loneliness [150]. This suggests that leisure travel may contribute to improved cognitive and psychosocial health by promoting social interactions, expanding social networks, and encouraging meaningful interpersonal connections.

Although several studies identified factors such as nutrition education, physical activity, and psychosocial support as being associated with improved nutritional knowledge, functional outcomes, and perceived quality of life, the evidence base remains limited and heterogeneous. Importantly, a clear distinction must be made between observational and interventional evidence. The majority of included studies were observational in nature and therefore can only suggest associations rather than causal effects. Only a small number of studies (n = 2) employed interventional designs, primarily pre–post- or quasi-experimental approaches, which generally reported improvements in outcomes such as nutritional knowledge. However, these studies were often limited by small sample sizes, short intervention durations, absence of control groups, and moderate risk of bias, thereby reducing the certainty of the evidence. Furthermore, effect sizes were inconsistently reported, and where available, the magnitude of change was modest and largely confined to intermediate outcomes (e.g., knowledge), with limited evidence for sustained improvements in clinical or functional endpoints. Therefore, while these findings suggest potential benefits of targeted interventions, the current evidence should be interpreted with caution. There is a critical need for rigorously designed randomized controlled trials with adequate follow-up to establish the effectiveness, scalability, and sustainability of such strategies in Sub-Saharan African contexts.

Overall, the methodological quality of the included studies was moderate–high, with just a few studies demonstrating low quality (Table 6, Table 7, Table 8 and Table 9). The majority of cross-sectional studies met key criteria such as clearly defined inclusion criteria, appropriate measurement of exposures and outcomes, and use of suitable statistical analyses. However, a consistent limitation across these studies was the inadequate identification and adjustment for confounding variables, with many domains rated as unclear due to insufficient reporting. Cohort and longitudinal studies generally exhibited stronger methodological rigor, particularly in the validity of exposure and outcome measurement and the use of appropriate analytical approaches. Nonetheless, several studies were limited by incomplete follow-up data and insufficient reporting on strategies to address missing data, which may introduce bias.

The quasi-experimental and pre–post-studies were of moderate quality, primarily due to the absence of control groups and limited repeated measurements, restricting causal inference. The qualitative study demonstrated high methodological quality overall, although researcher reflexivity was not clearly reported. Across all study designs, a recurring issue was limited reporting transparency, leading to multiple domains being classified as unclear. Therefore, while the overall body of evidence can be considered moderate in quality, the certainty of findings is constrained by methodological limitations, predominance of cross-sectional designs, and inconsistent handling of confounding factors.

This systematic review has limitations that should be considered when interpreting the findings presented in Table 1, Table 3 and Table 5. First, a formal protocol was not prospectively registered, which may limit transparency and reproducibility. Differences in search strategies across databases that were necessitated by platform-specific constraints may have affected the completeness of study retrieval. The evidence base was largely dominated by cross-sectional study designs, which restrict the ability to draw causal inferences regarding the relationships between nutritional status, aging-related challenges, and healthy aging outcomes. Consequently, associations observed across studies, such as those between malnutrition and frailty, food insecurity and functional disability, or nutritional status and cognitive decline cannot be interpreted as directional, because reverse causation remains possible. The broad scope of this review, encompassing nutritional status, age-related conditions, and intervention strategies, introduced conceptual and methodological heterogeneity, limiting the depth of synthesis within individual domains. The limited availability of longitudinal evidence further constrains understanding of nutritional and functional trajectories among older adults in Sub-Saharan Africa.

A second key limitation concerns the substantial methodological heterogeneity across the included studies, particularly those summarized in Table 2 and Table 4. Nutritional status and aging-related challenges were assessed using a wide array of instruments, including anthropometric measures, the Mini Nutritional Assessment and its short form, frailty indices, grip strength, gait speed, and self-reported functional indicators. Differences in measurement tools, diagnostic thresholds, and outcome definitions limit direct comparability across studies and likely account for some of the wide variation in reported prevalence of malnutrition, frailty, and disability across countries and settings. This lack of standardization constrains synthesis.

Finally, variability in study populations, geographical coverage, and evidence on interventions limits the generalizability and practical implications of the findings. Hospital-based studies and research involving specific clinical subgroups reported substantially higher burdens of malnutrition and aging-related challenges than community-based studies (Table 1 and Table 3), potentially overestimating population-level prevalence. In addition, the evidence base was concentrated in a small number of countries, with underrepresentation of several regions and of adults aged 80 years and above. Importantly, studies summarized in Table 5 were largely observational with few rigorously evaluated nutrition-specific interventions and frequent reliance on self-reported outcomes, introducing potential recall and reporting bias.

## 5. Conclusions

Malnutrition was the commonest nutrition-related challenge in the older Sub-Saharan African adults. In terms of setting, community-based studies reported that approximately 30–65% of the older adults were either malnourished or at risk of malnutrition, whereas hospital-based studies reported markedly higher burdens, with malnutrition prevalence exceeding 70% in some settings. Frailty emerged as the predominant age-related challenge, with prevalence ranging around 10–60%. These prevalences varied markedly across studies, reflecting differences in assessment tools, study settings, and population characteristics. Other common challenges included sarcopenia, reduced muscle strength, functional disability, cognitive impairment, depressive symptoms, and dysphagia, all of which were closely related to poor nutritional status, food insecurity, multimorbidity, and reduced quality of life. Geographically, most of the studies were conducted in South Africa, Ghana, and Nigeria, highlighting great disparities in terms of representation. Limited evidence obtained predominantly from observational studies showed that factors including nutrition education, physical activity, and psychosocial support may improve nutritional knowledge, functional outcomes, and perceived quality of life. Future studies should therefore focus on well-powered interventional studies employing a randomized controlled study design that are context-specific and nutrition-sensitive and that are aimed at improving nutritional and geriatric outcomes as a strategy to promote healthy aging in older adults from Sub-Saharan Africa.

## Figures and Tables

**Table 1:** Nutritional status and its associated factors in older Sub-Saharan African adults.

Authors Names, Year	Country of Study	Study Design	Sample Size (n)	Participants Characteristics	Objective of Study	Outcome of Assessment	Findings
Abdu et al. [38]	Ethiopia	Quantitative cross-sectional study	592 participants	Older adults aged ≥ 65 years	To assess the nutritional status and predictors of malnutrition among older adults (≥65 years) residing in Harari region, Eastern Ethiopia.	Weight, height, body mass index, and mid-upper arm circumference	51.7% and 15.7% were at risk of being malnourished and malnourished, respectively. Predictors of malnutrition included being of rural resident, having chronic pain, previous hospitalization, and inability to cover personal expenses.
Ahmed et al. [39]	Ethiopia	Cross-sectional study	262 participants	Older adults aged ≥ 50 years and living with heart failure	To determine the magnitude and associated factors of undernutrition among older adults living with heart failure in the hospital setting in Northwest Ethiopia.	Body mass index, weight loss, mid-arm and calf circumferences	28.6% of the participants were undernourished. Having severe heart failure, living with comorbidities, being on loop diuretic treatment, and of rural resident were associated with being undernourished.
Awuviry-Newton et al. [21]	Ghana	Cross-sectional study	4446 participants	Older adults aged > 50 years	To examine the associations between food insecurity and functional disability among older people in Ghana and the roles of gender and physical activity (PA) in the relationship.	Food insecurity; sufficiency of food intake, hunger and functional decline	Prevalence of older adults with food insecurity and experiencing hunger was 127% and 135%, respectively. They were also more likely to have a decline in their functional status.
Corso et al. [40]	Ghana and South Africa	Longitudinal study	6113 Ghanaian and 3623 South African participants	Older adults aged > 60 years	To examine the association between nutritional status and changes in cognition and grip strength among older Ghanaian and South African adults.	Nutritional status	48.9% (Ghanaians) and 31.3% (South Africans) were at risk of being malnourished. Less than 10% (Ghanaians) and 3.6% (South Africans) were malnourished. Malnourished older adults had lower cognitive scores and handgrip strength.
Dufe Turkson et al. [41]	Lesotho	Cross-sectional study	300 participants	Older adults aged ≥ 65 years	To assess the nutritional status of community-dwelling elderly in Lesotho and factors associated with malnutrition.	Weight, height, mid-arm circumference, and calf circumference	66.0% of the participants were at risk of malnutrition, and 14.6% (n = 44) were malnourished. Having psychological stress, being acutely ill, having perception of nutritional problems, and poor health status were factors associated with developing malnutrition.
Farombi et al. [42]	Nigeria	Cross-sectional study	99 participants	Older adults aged > 60 years	To assess the prevalence of malnutrition, burden of micronutrient deficiencies, and its influence on gender among the elderly population attending a geriatric clinic in Ibadan.	Malnutrition assessment	27.3% of the participants were at risk of being malnourished. Prevalence of vitamin A and D deficiencies was 10% and 1%, respectively, and was prominent in those aged 70–79 years. Males exhibited a greater burden of vitamin A insufficiency than females
Habumugisha et al. [43]	Rwanda	Cross-sectional study	334 participants	Older adults aged ≥ 55 years	To assess animal-sourced food (ASF) consumption and its associations with protein intake and muscle mass of older adults.	Dietary intake and anthropometric indices	56% of the participants consumed ASF. ASF intake was associated with high protein intake in both men and women. However, increased muscle mass was observed in only women.
Isong et al. [44]	Nigeria	Cross-sectional study	85 participants	Older adults aged ≥ 65 years	To evaluate the nutritional indices and associated risk factors of malnutrition among this population.	Nutritional status	58.2% of older adults were at risk of malnutrition, and 9.1% had poor nutritional status. Being dependent (most of the dependents are institutionalized) is a risk factor for developing malnutrition.
Jésus et al. [45]	Central African Republic (CAR) and Republic of Congo (ROC)	Cross-sectional population-based study	1055 participants	Older adults aged ≥ 65 years old	To determine the prevalence of undernutrition and obesity and the factors associated with both in adults over 65 years of age living in ROC and CAR.	Weight, height, body mass index, waist circumference, and diet	Prevalence of undernutrition was 19.2%, which was higher in CAR. It was positively associated with increased age, occupation as farmer/breeder, smoking, and low sugar consumption. Prevalence of obesity was 8.8%, which was higher in ROC and associated with female sex.
Junaid et al. [46]	Nigeria	Cross-sectional study	96 participants living with type 2 diabetes mellitus (T2DM) and 96 participants without T2DM	Older adults aged ≥ 60 years with T2DM	To determine the prevalence of malnutrition and associated factors in the elderly with T2DM.	Nutritional status	Malnutrition was highest among older adults with T2DM (7.3% vs. 0), and a high proportion of those with T2DM were underweight (16.7% vs. 4.2%); overweight and obese (58.3% vs. 24.0%) compared to those without T2DM group.
Mabiama et al. [47]	Cameroon	Cross-sectional study	599 participants	Older adults aged ≥ 60 years	To assess the nutritional status, health status, and associated sociodemographic factors among elderly in Cameroon.	Weight, height, body mass index (BMI), waist circumference (WC) and mid-upper arm circumference (MUAC)	19.7% were undernourished, 24.9% were overweight, and 17.5% were obese. Undernutrition was associated with MUAC < 24 cm, being of male gender, and uneducated, while obesity was associated with polypathology, being inactive and being an urban resident.
Mendham et al. [48]	South Africa	Cross-sectional study	122 participants	Black South African women between the ages of 60 and 85 years	To investigate the prevalence and correlates of sarcopenic obesity and its diagnostic components in older SA women from a low-income setting.	Food security, grip strength, appendicular skeletal muscle mass (ASM) and body mass index (BMI)	Prevalence of sarcopenia was 27.9%, which comprised sarcopenia without obesity (3.3%) and sarcopenic obesity (24.6%). Being food secured, low physical activity and having chronic inflammation were factors associated with developing sarcopenic obesity.
Mphwanthe et al. [49]	Malawi	Cross-sectional study	315 older adults	Older adults aged ≥ 60 years	To undertake malnutrition screening among older adults at hospital admission and examine the relationship between nutritional status and a range of health and socioecological factors.	Risk of malnutrition	39.7% and 40.3% of the participants were at risk of malnutrition development and malnourished, respectively. Factors including poor appetite, severe functional impairment, polypharmacy, and severe food insecurity were associated with increased risk of malnutrition development.
Naidoo et al. [50]	South Africa	Cross-sectional study	984 participants	Older adults aged ≥ 60 years	To identify determinants of nutritional risk among community-dwelling adults in KwaZulu-Natal, South Africa.	Nutritional risk	43.4% and 5.5% of the participants were at risk of developing malnutrition and being malnourished, respectively. Being male, having a monthly household income of ≤R1600 per month, and being depressed were factors associated with increased risk of malnutrition development.
Okoro et al. [51]	Nigeria	Cross-sectional study	122 participants	Patients aged ≥ 60 years	To assess the prevalence of malnutrition among elderly hospitalized patients in a Nigerian tertiary healthcare setting.	Anthropometric measures and nutritional status	71.3% of the participants were classified as malnourished, and 22.1% at risk of malnutrition.
Olawumi et al. [52]	Nigeria	Cross-sectional study	348 participants	Patients aged ≥ 60 years	To determine the nutritional status and its association with the morbidity patterns of elderly patients.	Anthropometric measurements and nutritional status	25.3% and 56.6% of the participants were malnourished and at risk of developing malnutrition, respectively. Prevalence of multimorbidity was 74.4%. Factors including advanced age, underweight, low educational status, chronic respiratory diseases, and physical inactivity were associated with malnutrition development.
Tesfaye et al. [53]	Ethiopia	Cross-sectional study	157 inpatients	Inpatients aged ≥ 60 years	To quantify the prevalence of malnutrition in older patients on inpatient admission and determine its associated factors.	Nutritional status	81% and 17% of the participants were malnourished and at risk for malnutrition development, respectively. Being a rural resident, having self-reported financial dependence for expenses, and having partial dependence in functional autonomy on admission were factors associated with the risk of malnutrition development.

Key: ≥, greater than or equal to; >, greater than.

**Table 2:** Classification of malnutrition prevalence by setting and assessment tool.

Study	Country	Setting	Participant Characteristics	Outcome of Assessment	Tool Used	Prevalence
Abdu et al. [38]	Ethiopia	Community	Older adults aged ≥ 65 years	Malnutrition; at risk	BMI, MUAC	15.7% malnourished; 51.7% at risk
Ahmed et al. [39]	Ethiopia	Hospital	Heart failure patients ≥ 50 years	Undernutrition	BMI, anthropometry	28.6% malnourished
Corso et al. [40]	Ghana and South Africa	Community	Older adults aged ≥ 60 years	Malnutrition; at risk	MNA-SF	<10% malnourished; 48.9% at risk in Ghana and 3.6% malnourished; 31.3% at risk in South Africa
Dufe Turkson et al. [41]	Lesotho	Community	Older adults aged ≥ 65 years	Malnutrition; at risk	Anthropometry	14.6% malnourished; 66.0% at risk
Farombi et al. [42]	Nigeria	Hospital	Older adults aged ≥ 60 years	Nutritional risk	MNA	27.3% at risk
Isong et al. [44]	Nigeria	Institutional	Older adults aged ≥ 65 years	Malnutrition; at risk	MNA-SF	9.1% malnourished; 58.2% at risk
Jésus et al. [45]	Central African Republic and Republic of Congo	Community	Older adults aged ≥ 65 years	Undernutrition	BMI, WC	19.2% malnourished
Junaid et al. [46]	Nigeria	Clinical	Older adults aged ≥ 60 years (T2DM)	Malnutrition	MNA-SF	7.3% malnourished
Mabiama et al. [47]	Cameroon	Community	Older adults aged ≥ 60 years	Undernutrition	BMI, MUAC	19.7% malnourished
Mphwanthe et al. [49]	Malawi	Hospital	Older adults aged ≥ 60 years	Malnutrition; at risk	MNA-SF	40.3% malnourished; 39.7% at risk
Naidoo et al. [50]	South Africa	Community	Older adults aged ≥ 60 years	Malnutrition; at risk	MNA-SF	5.5% malnourished; 43.4% at risk
Okoro et al. [51]	Nigeria	Hospital	Older adults aged ≥ 60 years	Malnutrition; at risk	Anthropometry	71.3% malnourished; 22.1% at risk
Olawumi et al. [52]	Nigeria	Hospital	Older adults aged ≥ 60 years	Malnutrition; at risk	Anthropometry	25.3% malnourished; 56.6% at risk
Tesfaye et al. [53]	Ethiopia	Hospital	Older adults aged ≥ 60 years	Malnutrition; at risk	MNA	81.0% malnourished; 17.0% at risk

Key: BMI, body mass index; MUAC, mid-upper arm circumference; WC, waist circumference; MNA, Mini Nutritional Assessment; MNA-SF, Mini Nutritional Assessment, Short Form; and ≥, greater than or equal to.

**Table 3:** Age-related challenges in older Sub-Saharan African adults focusing on functional status.

Authors Name, Year	Country of Study	Study Design	Sample Size (n)	Participants Characteristics	Objective of Study	Outcome of Assessment	Findings
Adebusoye et al. [54]	Nigeria	Cross-sectional study	642 participants	Older adults ≥ 60 years	To determine the prevalence and factors associated with sarcopenia among persons aged 60 years and above at a geriatric center in Nigeria.	Low muscle mass and low physical performance	Prevalence of sarcopenia was 5.4% and was higher among females. Low muscle mass and decreased gait speed were found in 10.9% and 36.1% of the participants, respectively. Age, having no formal education, malnutrition, and female gender were the predictors of sarcopenia.
Adjaye-Gbewonyo et al. [55]	Ghana, South Africa	Longitudinal study	4199 Ghanaian and 3174 South African adults	Older adults aged ≥ 50 years	To examine the association between urban–rural residence across the life course and the risk of depression in later life in Ghana and South Africa.	Depression	The rates of depression in the samples based on type of adulthood residence were 7.1% for urban and 8.1% for rural in Ghana, and 4.8% for urban and 3.8% for rural in South Africa.
Ambagtsheer and Moussa [56]	Côte d’Ivoire	Prospective study	860 participants	Older adults aged ≥ 50 years	To explore the relationship between frailty and health service utilization and expenditure within Côte d’Ivoire.	Frailty	60.0% and 22.8% of the participants were frail and prefrail, respectively. Increased health service utilization was associated with frailty for hospital attendance, traditional practitioners, and self-medication. Frailty was also associated with aggregate consulting costs and medications.
Asiamah et al. [57]	Ghana	Cross-sectional study	1484 participants	Community-dwelling older adults aged ≥ 50 years	To examine the association between frailty and functional difficulty between low and higher-income groups and between older men and women in these income groups.	Frailty and functional difficulty (performance of self-care)	Frailty was indeed positively associated with functional difficulty in both participants though higher in the low-income group. Women reported greater values in both samples.
Awuviry-Newton et al. [21]	Ghana	Cross-sectional study	4446 participants	Older adults > 50 years	To examine the associations between food insecurity and functional disability among older people in Ghana, and the roles of gender and physical activity in the relationship.	Food insecurity; sufficiency of food intake and hunger. Functional decline using 6 domains (cognition, mobility, self-care, getting along, life activities, and participation in society)	Higher prevalence of functional disability among those who consumed insufficient food (15.3%) and experienced hunger (15.7%) compared to those without food insecurity. Gender and physical activity were associated with both variables.
Baiyewu et al. [58]	Nigeria	Longitudinal study	4425 participants, but 135 of the original were recruited for re-evaluation	Older adults aged ≥ 65 years	To revisit these subjects and evaluate them for the presence of cognitive and functional impairment and assign diagnoses as relevant, as well as determine factors that mitigate development of dementia and cognitive impairment and thus promote healthy aging.	Dementia and cognitive impairment	21% were diagnosed with dementia, 18 % had Alzheimer’s disease, 32.6 % had diagnosis of mild cognitive impairment, and 45.9 % were cognitively normal. Cognitive score, diastolic blood pressure, and regular alcohol use were associated with 20-year prevalence of cognitive impairment when age, sex, and education were adjusted.
Bell et al. [59]	South Africa	Comparative study	44 participants	Mean age of 80 years (standard deviation ± 6.54)	To compare self-perceived and clinical presentation of eating and swallowing abilities among a portion of elderly residents to enhance management of the residential care population.	Oropharyngeal dysphagia assessment and swallowing ability	Food sticking in the throat was the most common self-reported swallowing difficulty (45%), with high Eating Assessment Tool-10 scores also reported for swallowing pills requiring extra effort (50%).
Biritwum et al. [60]	Ghana and South Africa	Cross-sectional study	Ghana (4305 respondents) South Africa (3832)	Older adults aged > 50 years	To provide estimates of the prevalence of frailty and disability in older adult populations and to examine their relationship with socioeconomic factors in six countries.	Frailty Index	Both frailty and disability increased with age for both countries and was higher for women.
Brennan-Olsen et al. [61]	Ghana and South Africa	Cross-national study	Ghana n = 1640 South Africa n = 870	Older adults ≥ 65 years	To investigate associations between two functional measures of sarcopenia, grip strength and gait speed (GS), with functional disability in adults from six low- and middle-income countries.	Functional disability, sarcopenia (gait speed and grip strength)	Individually and combined, low grip strength and slow gait speed were associated with worse functional disability scores, independent of comorbidities, low education, and low wealth.
Bristow et al. [62]	Tanzania	Cross-sectional study	145 participants	Older adults aged ≥ 50 years	To investigate the prevalence of frailty in an older HIV+ population in Sub-Saharan Africa (SSA) and screening and diagnostic tools to identify frailty in SSA.	Frailty	Frailty prevalence was overall low, ranging from 0.7% to 2.8% depending on the tool used. Frailty was associated with female sex, marital status, and age, with weight loss being the most common feature.
Corso et al. [40]	Ghana and South Africa	Longitudinal study	6113 Ghanaian and 3623 South African records datasets	Older adults aged > 60 years	To examine the association between nutritional status and changes in cognition and grip strength among older Ghanaian and South African adults.	Nutritional status, cognition performance, handgrip strength	Ghana recorded a cognition z-score of 58.0 and a mean handgrip strength of 23.4 kg, whilst South Africa recorded a varied score of lower mean cognition (49.4) but a higher mean handgrip strength (36.4 kg). There is a confirmation of a positive association between nutritional status, cognition performance, and handgrip strength.
Davidson et al. [63]	Tanzania	Prospective observational study	308 participants	Older adults aged ≥ 60 years	To measure the prevalence of frailty amongst older people admitted to hospital in Tanzania and to explore their demographic and clinical characteristics.	Frailty	Overall, frailty prevalence was higher, with 66.6% of participants classified as frail using the Clinical Frailty Scale and 57.0% identified as frail using the Frailty Phenotype. Participants with frailty were significantly older, with low levels of education and literacy, greater disability and comorbidity, poorer cognition, and increased levels of delirium.
Dzando and Moussa [64]	Côte D’Ivoire	Cross-sectional study	1017 participants	Older adults aged ≥ 50 years	To investigate the relationship between frailty and subjective life expectancy in Sub-Sahara Africa.	Frailty and subjective life expectancy	59.3% of participants were frail, and frailty was associated with low subjective life expectancy, with frail individuals reporting up to 3.7 fewer years than non-frail individuals.
Dzando and Moussa [65]	Côte D’Ivoire	Cross-sectional study	1017 participants	Older adults aged ≥ 50 years	To examine the health deficits that contribute to frailty among older people in Côte d’Ivoire.	Frailty	59.3% were classified as frail, with a higher prevalence in females (70.5%). Frailty increased with age, peaking at 74.6% among the oldest group.
Dzando et al. [66]	Uganda	Cross-sectional study	444 participants	Older adults aged ≥ 50 years	To investigate the contributions of specific frailty domains to quality of life (QoL) and whether these associations differ by sex remain poorly understood.	Frailty	The mean overall frailty score was 0.27 (SD = 0.20). Increased levels of overall frailty were significantly associated with low QoL.
Ede et al. [67]	Nigeria	Qualitative study	14 participants	Older adults aged 60–89 years	To explore how older people in Southeastern Nigeria interpret and express their sexual behavior in later life.	Sexual behavior	Later-life sexual behavior was diverse, with older adults adapting toward companionship and affection, influenced by modifiable interpersonal factors that present opportunities for intervention.
Gatimu et al. [68]	Ghana	Cross-sectional study	4135 participants	Older adults aged ≥ 50 years	To determine the prevalence and determinants of diabetes among adults aged 50 years and above in Ghana.	Prevalence of diabetes	The weighted prevalence of diabetes was 3.95%, with the prevalence being insignificantly higher in females than males. The determinants of diabetes were low physical activity and obesity in women, and then old age and university, secondary and primary education in men
Gray et al. [69]	Tanzania	Observational cohort study	Cohort (n = 1198) and the stratified sample (n = 296)	Older adults aged ≥ 70 years	To identify frailty and its outcomes in older people in rural Tanzania.	Frailty Index	A higher Frailty Index score was significantly correlated with greater age, never having attended school, falls, mortality, and dependency in activities of daily living.
Gyasi et al. [70]	Ghana	Cross-sectional study	1201 participants	Older adults aged ≥ 50 years	To examine the cross-sectional association of bodily pain with emotional pain in a representative sample from Ghana and the potential mediators of this association.	Bodily pain severity and emotional pain	In total, 30.1% of the individuals reported emotional pain. The severity of bodily pain was dose-dependently and incrementally associated with higher odds for emotional pain.
Gyasi et al. [71]	Ghana	Cross-sectional study	1201 participants	Older adults aged ≥ 50 years	To examine the association between subjective sleep problems and global SRH among older adults in Ghana and to explore the sex-based mediating effect of pain interference in this association.	Self-rated health (SHR) and sleep	Increased sleep problems were independently associated with worse global SRH outcomes in the overall sample.
Gyasi et al. [72]	Ghana	Cross-sectional study	1201 participants	Older adults aged ≥ 50 years	To examine the association of diabetes mellitus (DM) with functional limitations (FL) in older adults and to identify potential factors influencing this association.	Functional limitation outcome and diabetes mellitus exposure	The prevalence of DM and FL was 10.1% and 36.1%, respectively, with physical activity mediating 40.39% of the DM-FL association
Gyasi and Philips [73]	Ghana	Cross-sectional study	1200 participants	Older adults aged ≥ 50 years	To examine the association between self-rated health (SRH) and functional decline (FD) in older Ghanaian cohorts.	Functional decline (FD) and self-rated health (SRH)	Functional decline was reported by 23% of men and 34% of women. Poorer SRH strongly predicted high functional disability in both genders, with stronger effects among men.
Heward et al. [74]	Tanzania	Longitudinal study	Cohort (n = 473); follow-up (n = 327)	Older adults aged ≥ 65 years	To detail the prevalence of cognitive decline and identify potentially modifiable risk factors for decline over a two-year period in a community-dwelling, rural cohort of individuals aged 65 years or over in Northern Tanzania.	Cognitive function functional disability, hypertension, and grip strength (as a measure of frailty)	Among 327 participants with baseline and follow-up data, 50 experienced cognitive decline over two years. Cognitive decline was independently associated with low baseline grip strength, female sex, and depression at follow-up.
Kasa et al. [22]	Ethiopia	Quasi-experimental study	68 participants	Older adults aged ≥ 60 years	To assess the initial correlations among frailty, nutritional status, depression, and QOL (quality of life) in a group of older people in Ethiopia.	Frailty	The mean frailty score was 7.3, with increased frailty associated with poor nutritional status and increased levels of depression. High frailty was consistently linked to low quality of life across all domains.
Lewis et al. [75]	Tanzania	Cross-sectional study	235 participants	Older adults aged ≥ 60 years	To operationalize the frailty phenotype in a rural Tanzanian population of older community-dwelling adults.	Frailty	The prevalence of frailty ranged from 9.3% (196 participants) to 11.2% (235). Frailty was more common at older ages and was associated with poor self-rated health and depressive symptoms.
Lewis et al. [76]	Tanzania	Cross-sectional study	236 participants	Older adults aged ≥ 60 years	To investigate the prevalence of frailty by comprehensive geriatric assessment for older community-dwelling adults living in rural Northern Tanzania.	Frailty	91 individuals were deemed frail. Overall prevalence of frailty is 19.18%, which increased with age, reaching 44.7% in adults aged ≥ 80 years.
Ó Breasail et al. [77]	Gambia	Pilot study	Rural (n = 209) and urban (n = 101)	Older adults aged ≥ 55 years	To compare bone and muscle health in older adults living in rural and urban Gambia.	Grip strength (sarcopenia) and osteoporosis	Osteoporosis at either the femoral neck or total hip was more prevalent in urban men (20% vs. rural 10%) and rural women (45% vs. urban 31%). Sarcopenia was also high in rural participants (men: 30% vs. 18%; women: 18% vs. 15%).
Mabiama et al. [78]	Cameroon	Cross-sectional study	599 individuals	Older adults aged ≥ 60 years	To assess the factors associated with depressive syndrome (DS) and cognitive impairment (CI) among older adults in Cameroon.	Depressive syndrome (DS) and cognitive impairment (CI)	DS affected 14.5% of the population and the CI was 21.4%. Variations were found according to sociodemographic, health, and nutritional characteristics.
Mbabazi et al. [79]	Uganda	Cross-sectional study	749 participants	Older adults aged ≥ 60 years	To determine the prevalence and factors associated with frailty among older adults with and without HIV in an urban outpatient clinic in Kampala, Uganda.	Frailty	Older adults living with HIV had a similar prevalence of frailty (15.1% vs. 13.5%) and prefrailty (45.2% vs. 43.1%) compared to older adults without HIV. Frailty was associated with older age, female sex, having no partner, being underweight, presenting food insecurity, and depressive symptoms.
Mwangala et al. [80]	Kenya	Cross-sectional study	440 participants	Older adults aged ≥ 50 years	To document the prevalence and correlates of frailty among older adults living with HIV (OALWH) and their uninfected peers and investigate HIV status as an independent predictor of frailty.	Frailty	The prevalence of frailty was higher among OALWH (24%) than their uninfected peers (13%). HIV seropositivity was not independently associated with frailty.
Smith et al. [81]	Ghana and South Africa	Cross-sectional study	Ghana n = 1975; South Africa n = 1484	Older adults aged ≥ 65 years	To investigate the association between multimorbidity and sarcopenia in a sample of older adults from six low- and middle-income countries (China, Ghana, India, Mexico, Russia, and South Africa).	Physical multimorbidity and sarcopenia (skeletal muscle mass, handgrip strength, and gait speed)	The prevalence of 1 and ≥2 chronic physical conditions was 26.2% and 60.2%, respectively, while 12.0% had sarcopenia, and 7.8% severe sarcopenia. Those with sarcopenia were older and had low levels of education, wealth, and physical activity. They also had a high prevalence of arthritis, chronic back pain, edentulism, hearing problems, visual impairment, and multimorbidity.
Smith et al. [82]	Ghana and South Africa	Cross-sectional study	Ghana (n = 1975); South Africa (n = 1484)	Older adults aged ≥ 65 years	To investigate the association between sleep duration and sarcopenia among adults aged ≥ 65 years from five low- and middle-income countries (China, Ghana, India, Russia, and South Africa).	Sarcopenia and sleep duration	Long sleep duration was associated with increased odds of sarcopenia and severe sarcopenia in low- and middle-income countries, particularly in females.
Zimba Kalula et al. [83]	South Africa	Cross-sectional study with a 12 m follow-up	837 participants (baseline); 632 participants (follow-up)	Community-dwelling older adults aged ≥ 65 years	To establish a rate for falls in older adults in South Africa.	History of falls	Rates of 26.4% and 21.9% for falls and of 11% and 6.3% for recurrent falls, respectively, were calculated at baseline and follow-up.

Key: ≥, greater than or equal to; >, greater than.

**Table 4:** Frailty prevalence by setting and assessment tool.

Study	Country	Setting	Outcome of Assessment	Tool Used	Prevalence
Ambagtsheer and Moussa [56]	Côte d’Ivoire	Urban and rural clusters	Frailty	30-item Frailty Index	Among the participants, 60.0% were frail, 22.8% prefrail and 17.2% robust.
Asiamah et al. [57]	Ghana	Community-dwelling older adults in low- and high-income groups	Frailty	15-item Chinese version of the Tilburg Frailty Indicator	Frailty in the low-income sample was higher (Mean = 6.23).
Biritwum et al. [60]	Ghana and South Africa	N/A	Frailty	40-item Frailty Index	Approximately 38% for both countries
Bristow et al. [62]	Tanzania	Rural setting going for follow-up	Frailty	Fried frailty phenotype (FFP), Clinical Frailty Scale (CFS), and Brief Frailty Instrument for Tanzania (B-FIT 2)	The prevalence of frailty by FFP was 2.758%. The prevalence of frailty using the B-FIT 2 and the CFS was 0.68%.
Davidson et al. [63]	Tanzania	Older adults admitted in Tanzanian hospitals in urban, sub-urban, and rural areas	Frailty	Clinical Frailty Scale and the Frailty Phenotype	Overall, frailty prevalence was higher, with 66.6% of participants classified as frail using the Clinical Frailty Scale and 57.0% identified as frail using the Frailty Phenotype
Dzando and Moussa [64]	Côte D’Ivoire	Urban	Frailty	30-item Frailty Index	59.3% of participants were frail
Dzando and Moussa [65]	Côte D’Ivoire	Urban	Frailty	30-item Frailty Index	59.3% of participants were frail
Dzando et al. [66]	Uganda	Community-dwelling adults	Frailty	15 items from the Frailty Instrument for Sub-Saharan Africa	The mean overall frailty score was 0.27
Gray et al. [69]	Tanzania	Rural	Frailty	40-item Frailty Index	
Kasa et al. [22]	Ethiopia	Community-dwelling	Frailty	Amharic version of the Tilburg Frailty Indicator	The mean frailty score was 7.3
Lewis et al. [75]	Tanzania	Community-dwelling adults	Frailty	Frailty phenotype	The prevalence of frailty ranged from 9.3% (196 participants) to 11.2% (235)
Lewis et al. [76]	Tanzania	Community-dwelling adults	Frailty	Comprehensive geriatric assessment	91 individuals were deemed frail. Overall prevalence of frailty is 19.18%
Mbabazi et al. [79]	Uganda	Urban outpatient clinic	Frailty	Frailty phenotype	PWH had a similar prevalence of frailty (15.1% vs. 13.5%) and prefrailty (45.2% vs. 43.1%) compared to PWOH
Mwangala et al. [80]	Kenya	HIV-specialized clinics	Frailty	Reported Edmonton Frail Scale	The prevalence of frailty was higher among older adults living with HIV/AIDS (24%) than their uninfected peers (13%)

Key: N/A, not available.

**Table 5:** Strategies to promote healthy aging in older adults.

Authors Name, Year	Country of Study	Study Design	Sample Size	Participants Characteristics	Objective of Study	Outcome of Assessment	Findings
Atuhairwe et al. [84]	Uganda	Cross-sectional study	440 participants	Older adults > 50 years living with HIV	To investigate the relationship between perceived quality of life (QoL) and lifestyle factors among older adults living with HIV (OALWHIV) in Uganda.	Perceived QoL before and after the start on antiretroviral medication	Before initiating ART, 48% of people living with HIV (PLHIV) perceived their quality of life as normal, and after starting ART, 65% of PLHIV perceived their quality of life as normal.
Alaazi et al. [85]	Ghana	Cross-sectional study	603 participants (slum 302; non slum 301)	Older adults aged ≥ 60 years	To explore and compare the quality of life (QoL) of older slum and non-slum dwellers in Ghana; and determine the extent of QoL disparities between slum and non-slum older adults.	Quality-of-life assessment	Female sex, older age, lack of regular income, and being ill were associated with significantly lower physical QoL scores.
Belachew et al. [86]	Ethiopia	Cross-sectional study	845 participants	Older adults aged ≥ 60 years	To examine the prevalence and predictors of healthy aging among community-dwelling older adults living in Bahir Dar, Ethiopia.	Healthy aging status	The overall prevalence of healthy aging was 36.7% and highest in the 60–69 age group (72.6%).
Dzando et al. [66]	Uganda	Cross-sectional study	444 participants	Older adults aged ≥ 50 years	To investigate the contributions of specific frailty domains to quality of life (QoL) and whether these associations differ by sex remain poorly understood.	Quality-of-life assessment	The mean QoL score was 3.76. High levels of overall frailty were significantly associated with low QoL.
Govender and De Jongh [87]	South Africa	Cross-sectional study	70 participants	Older adults aged 65–98 age range	To determine the psychological, communication-related, and social impact of the hearing impairment on the quality of life in a sample of elderly participants residing in retirement homes.	Perceived impact of hearing loss on quality of life	51% (n = 31) of 60 participants perceived themselves as having a hearing problem. Of the total sample, (n = 70), 74% (n = 36) reported that the hearing problem negatively affects their lives.
Maniragaba et al. [88]	Uganda	Cross-sectional study	912 participants	Older adults aged ≥ 60 years	To examine the factors associated with older persons’ physical health in rural Uganda.	Physical health indicators	Older adults were more likely to have good physical health when household assets were controlled by themselves or their spouses rather than by their children, and when they engaged in physical activity compared to those who did not.
Oduro and Kissah-Korsah [89]	South Africa	Longitudinal study	440 adults	Older adults aged ≥ 50 years	To examine the nutritional wellness among aged persons living with HIV in Somkhele, South Africa.	Nutritional wellness	6 in 10 aged persons were HIV-infected (59.5%). Percentage of men with adequate nutrition was high (78.7%), and having nutritional wellness was low for aged persons who were infected by HIV.
Seid and Fentahun [90]	Ethiopia	Pre–post-study	263 participants	Community-dwelling older adults aged > 60 years	To evaluate the effect of behavioral model-guided nutritional counseling on the dietary intake and nutritional status of elders.	Nutritional knowledge, dietary intake, and body weight	Nutritional counseling increased the knowledge score from 7.58 (±1.05) to 11.6 (±1.37) (*p* < 0.001). The mean body weight and the body mass index did not change significantly after the intervention.

Key: ≥, greater than or equal to; >, greater than.

**Table 6:** JBI critical appraisal checklist for analytical cross-sectional studies [34].

Authors Name, Year	Inclusion Criteria	Subjects and Setting	Exposure Measured	Objective Criteria	Confounders Identified	Confounders Addressed	Outcome Measured	Statistical Analysis	Overall
Abdu et al. [38]	Yes	Yes	Yes	Yes	Unclear	No	Yes	Yes	Moderate
Ahmed et al. [39]	Yes	Yes	Yes	Yes	Yes	Unclear	Yes	Yes	High
Awuviry-Newton et al. [21]	Yes	Yes	Yes	Yes	Yes	Yes	Yes	Yes	High
Dufe Turkson et al. [41]	Yes	Yes	Yes	Yes	Unclear	No	Yes	Yes	Moderate
Farombi et al. [42]	Yes	Yes	Yes	Yes	Yes	Unclear	Yes	Yes	High
Habumugisha et al. [43]	Yes	Yes	Yes	Yes	Yes	Unclear	Yes	Yes	High
Isong et al. [44]	Yes	Yes	Yes	Yes	Unclear	No	Yes	Yes	Moderate
Jésus et al. [45]	Yes	Yes	Yes	Yes	Yes	Unclear	Yes	Yes	High
Junaid et al. [46]	Yes	Yes	Yes	Yes	Unclear	No	Yes	Yes	Moderate
Mabiama et al. [47]	Yes	Yes	Yes	Yes	Yes	Unclear	Yes	Yes	High
Mendham et al. [48]	Yes	Yes	Yes	Yes	Yes	Yes	Yes	Yes	High
Mphwanthe et al. [49]	Yes	Yes	Yes	Yes	Unclear	No	Yes	Yes	High
Naidoo et al. [50]	Yes	Yes	Yes	Yes	Yes	Yes	Yes	Yes	High
Okoro et al. [51]	Yes	Yes	Yes	Yes	Unclear	No	Yes	Yes	Moderate
Olawumi et al. [52]	Yes	Yes	Yes	Yes	Yes	Unclear	Yes	Yes	High
Tesfaye et al. [53]	Yes	Yes	Yes	Yes	Yes	Yes	Yes	Yes	High
Adebusoye et al. [54]	Yes	Yes	Yes	Yes	Yes	Yes	Yes	Yes	High
Asiamah et al. [57]	Yes	Yes	Yes	Yes	Yes	Unclear	Yes	Yes	High
Bell et al. [59]	Yes	Yes	Yes	Yes	Yes	Unclear	Yes	Yes	High
Biritwum et al. [60]	Yes	Yes	Yes	Yes	Yes	Yes	Yes	Yes	High
Brennan-Olsen et al. [61]	Yes	Yes	Yes	Yes	Yes	Yes	Yes	Yes	High
Bristow et al. [62]	Yes	Yes	Yes	Yes	Unclear	No	Yes	Yes	Moderate
Dzando and Moussa [64]	Yes	Yes	Yes	Yes	Unclear	No	Yes	Yes	Moderate
Dzando and Moussa [65]	Yes	Yes	Yes	Yes	Unclear	No	Yes	Yes	Moderate
Dzando et al. [66]	Yes	Yes	Yes	Yes	Yes	Unclear	Yes	Yes	High
Gatimu et al. [68]	Yes	Yes	Yes	Yes	Yes	Yes	Yes	Yes	High
Gyasi et al. [70]	Yes	Yes	Yes	Yes	Yes	Yes	Yes	Yes	High
Gyasi et al. [71]	Yes	Yes	Yes	Yes	Yes	Yes	Yes	Yes	High
Gyasi et al. [72]	Yes	Yes	Yes	Yes	Yes	Yes	Yes	Yes	High
Gyasi and Philips [73]	Yes	Yes	Yes	Yes	Yes	Yes	Yes	Yes	High
Lewis et al. [75]	Yes	Yes	Yes	Yes	Yes	Yes	Yes	Yes	High
Lewis et al. [76]	Yes	Yes	Yes	Yes	Yes	Yes	Yes	Yes	High
Mabiama et al. [78]	Yes	Yes	Yes	Yes	Yes	Unclear	Yes	Yes	High
Mbabazi et al. [79]	Yes	Yes	Yes	Yes	Yes	Yes	Yes	Yes	High
Mwangala et al. [80]	Yes	Yes	Yes	Yes	Yes	Yes	Yes	Yes	High
Smith et al. [81]	Yes	Yes	Yes	Yes	Yes	Yes	Yes	Yes	High
Smith et al. [82]	Yes	Yes	Yes	Yes	Yes	Yes	Yes	Yes	High
Zimba Kalula et al. [83]	Yes	Yes	Yes	Yes	Yes	Yes	Yes	Yes	High
Atuhairwe et al. [84]	Yes	Yes	Yes	Yes	Yes	Unclear	Yes	Yes	High
Alaazi et al. [85]	Yes	Yes	Yes	Yes	Yes	Yes	Yes	Yes	High
Belachew et al. [86]	Yes	Yes	Yes	Yes	Unclear	No	Yes	Yes	Moderate
Govender and De Jongh [87]	Yes	Yes	Yes	Yes	Yes	Yes	Yes	Yes	High
Maniragaba et al. [88]	Yes	Yes	Yes	Yes	Yes	Unclear	Yes	Yes	High

**Table 7:** JBI critical appraisal checklist for cohort studies [35].

Authors Name, Year	Similar Groups	Same Population	Exposure Similar	Exposure Valid	Confounders Identified	Confounders Addressed	Outcome-Free Baseline	Outcome Measured	Follow-Up Adequate	Follow-Up Complete	Missing Addressed	Statistical Analysis	Overall
Corso et al. [40]	Yes	Yes	Yes	Yes	Yes	Unclear	Yes	Yes	Yes	No	Unclear	Yes	Moderate
Adjaye-Gbewonyo et al. [55]	Yes	Yes	Yes	Yes	Yes	Yes	Yes	Yes	Yes	Yes	Yes	Yes	High
Baiyewu et al. [58]	Yes	Yes	Yes	Yes	Yes	Yes	Yes	Yes	Yes	Unclear	Unclear	Yes	High
Ambagtsheer and Moussa [56]	Yes	Yes	Yes	Yes	Unclear	No	Yes	Yes	Yes	No	Unclear	Yes	Moderate
Davidson et al. [63]	Yes	Yes	Yes	Yes	Yes	Yes	Yes	Yes	Yes	Yes	Yes	Yes	High
Heward et al. [74]	Yes	Yes	Yes	Yes	Yes	Unclear	Yes	Yes	Yes	No	Unclear	Yes	Moderate
Gray et al. [69]	Yes	Yes	Yes	Yes	Yes	Unclear	Yes	Yes	Yes	No	Unclear	Yes	Moderate
Oduro and Kissah-Korsah [89]	Yes	Yes	Yes	Yes	Yes	Unclear	Yes	Yes	Yes	No	Unclear	Yes	Moderate

**Table 8:** JBI critical appraisal checklist for quasi-experimental studies [36].

Authors Name, Year	Cause–Effect	Participants Similar	Treatment Similar	Control Group	Multiple Measures	Follow-Up Complete	Outcome Reliable	Statistical Analysis	Overall
Kasa et al. [22]	Yes	Yes	Yes	No	Unclear	Yes	Yes	Yes	High
Seid and Fentahun [90]	Yes	Yes	Yes	No	Yes	Yes	Yes	Yes	Moderate

**Table 9:** JBI critical appraisal checklist for qualitative research [37].

Authors Name, Year	Philosophy	Methodology	Methods	Data Collection	Representation	Reflexivity	Ethics	Analysis	Findings	Overall
Ede et al. [67]	Yes	Yes	Yes	Yes	Yes	Unclear	Yes	Yes	Yes	High

## Data Availability

Data is contained within the article.
